# International multicenter examination of MOG antibody assays

**DOI:** 10.1212/NXI.0000000000000674

**Published:** 2020-02-05

**Authors:** Markus Reindl, Kathrin Schanda, Mark Woodhall, Fiona Tea, Sudarshini Ramanathan, Jessica Sagen, James P. Fryer, John Mills, Bianca Teegen, Swantje Mindorf, Nora Ritter, Ulrike Krummrei, Winfried Stöcker, Juliane Eggert, Eoin P. Flanagan, Melanie Ramberger, Harald Hegen, Kevin Rostasy, Thomas Berger, Maria Isabel Leite, Jacqueline Palace, Sarosh R. Irani, Russell C. Dale, Christian Probst, Monika Probst, Fabienne Brilot, Sean J. Pittock, Patrick Waters

**Affiliations:** From the Clinical Department of Neurology (M. Reindl, K.S., M. Ramberger, H.H.), Medical University of Innsbruck, Innsbruck, Austria; Oxford Autoimmune Neurology Group (M.W., M. Ramberger, M.I.L., J.P., S.R.I., P.W.), Nuffield Department of Clinical Neurosciences, University of Oxford, United Kingdom; Brain Autoimmunity Group (F.T., S.R., R.C.D., F.B.), Kids Neuroscience Centre at Kids Research at the Children's Hospital at Westmead, Brain and Mind Centre, University of Sydney, New South Wales, Australia; Department of Neurology (J.S., J.P.F., J.M., E.P.F., S.J.P.), Mayo Clinic, Rochester, MN; Euroimmun Medizinische Labordiagnostika AG (B.T., S.M., N.R., U.K., W.S., C.P.), Lübeck, Germany; Institute for Quality Assurance (ifQ) affiliated to Euroimmun (J.E., M.P.), Lübeck, Germany; Paediatric Neurology (K.R.), Witten/Herdecke University, Children's Hospital Datteln, Datteln, Germany; and Department of Neurology (T.B.), Medical University of Vienna, Austria.

## Abstract

**Objective:**

To compare the reproducibility of 11 antibody assays for immunoglobulin (Ig) G and IgM myelin oligodendrocyte glycoprotein antibodies (MOG-IgG and MOG-IgM) from 5 international centers.

**Methods:**

The following samples were analyzed: MOG-IgG clearly positive sera (n = 39), MOG-IgG low positive sera (n = 39), borderline negative sera (n = 13), clearly negative sera (n = 40), and healthy blood donors (n = 30). As technical controls, 18 replicates (9 MOG-IgG positive and 9 negative) were included. All samples and controls were recoded, aliquoted, and distributed to the 5 testing centers, which performed the following antibody assays: 5 live and 1 fixed immunofluorescence cell-based assays (CBA-IF, 5 MOG-IgG, and 1 MOG-IgM), 3 live flow cytometry cell-based assays (CBA-FACS, all MOG-IgG), and 2 ELISAs (both MOG-IgG).

**Results:**

We found excellent agreement (96%) between the live CBAs for MOG-IgG for samples previously identified as clearly positive or negative from 4 different national testing centers. The agreement was lower with fixed CBA-IF (90%), and the ELISA showed no concordance with CBAs for detection of human MOG-IgG. All CBAs showed excellent interassay reproducibility. The agreement of MOG-IgG CBAs for borderline negative (77%) and particularly low positive (33%) samples was less good. Finally, most samples from healthy blood donors (97%) were negative for MOG-IgG in all CBAs.

**Conclusions:**

Live MOG-IgG CBAs showed excellent agreement for high positive and negative samples at 3 international testing centers. Low positive samples were more frequently discordant than in a similar comparison of aquaporin-4 antibody assays. Further research is needed to improve international standardization for clinical care.

Immunoglobulin (Ig) G antibodies to myelin oligodendrocyte glycoprotein (MOG-IgG) are found in adults and children who present with a spectrum of CNS features that include optic neuritis, acute disseminated encephalomyelitis (ADEM), myelitis, seizures, encephalitis, brainstem, and/or cerebellar involvement. In addition, the presence of MOG-IgG can discriminate these disorders from MS.^1^ Numerous studies have used different immunoassays for MOG-IgG detection, but it is now clear that native full-length human MOG as an assay substrate is crucial to make this clinical distinction. When measured using first generation assays (ELISA and Western blot), MOG-IgG are prevalent and have been identified in healthy individuals and patients with a wide variety of clinical presentations. Thus, their detection was initially considered to have little clinical utility. However, when measured by live cell-based assays (CBAs), an association between MOG-IgG antibodies and a non-MS demyelinating phenotype has been established. This understanding has driven the establishment of different variants of MOG-IgG assays with native MOG substrates in multiple centers worldwide. There are limited data on assay reproducibility between these centers. In this study, we compared the most frequently used assays for MOG-IgG detection, such as live and fixed immunofluorescence cell-based assays (CBA-IF),^2–17^ live flow cytometry cell-based assays (CBA-FACS),^4,18–27^ and ELISA.^28,29^

## Methods

### Patients and controls

**Figure 1:**
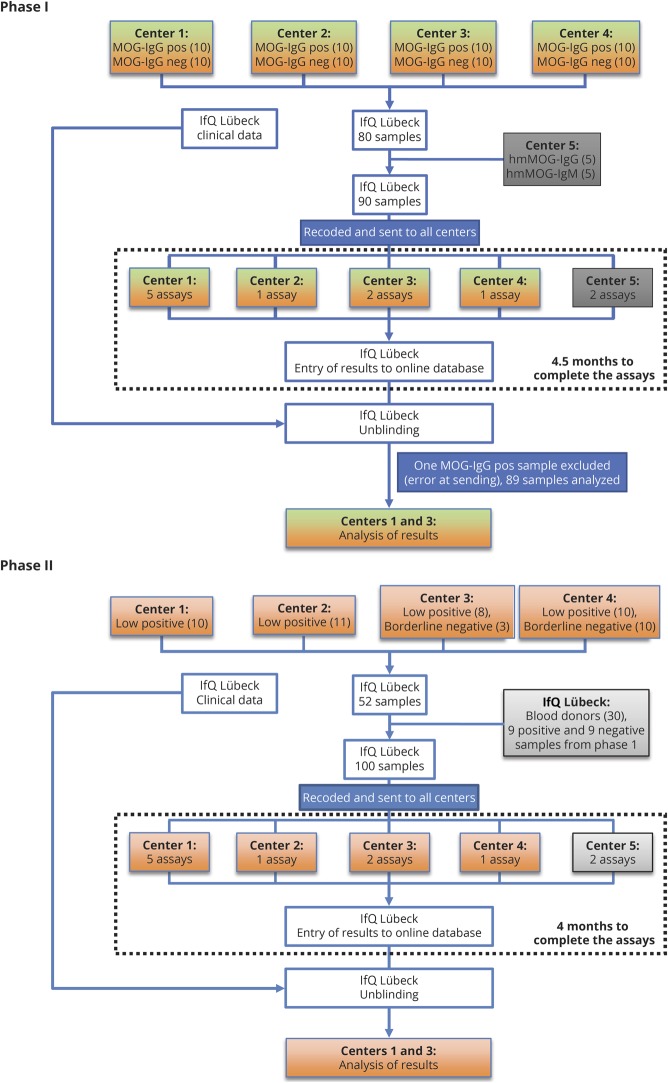
Flowchart showing phases I and II of this study Center 1 (Innsbruck) performed 5 assays (live CBA-IF MOG-IgG (H + L), live CBA-IF MOG-IgG(Fc), live CBA-FACS MOG-IgG(Fc), live CBA-IF MOG-IgM, and ELISA MOG-IgG); center 2 (Mayo Clinic) performed 1 assay (live CBA-FACS MOG-IgG1); center 3 (Oxford) performed 2 assays (live CBA-IF MOG-IgG (H + L) and live CBA-IF MOG-IgG1); center 4 (Sydney) performed 1 assay (live CBA-FACS MOG-IgG (H + L)), which was repeated twice; center 5 (Euroimmun) performed 2 assays (fixed CBA-IF MOG-IgG(Fc) and ELISA MOG-IgG(Fc)). CBA = cell-based assay; FACS = fluorescence-activated cell sorting; IF = immunofluorescence; IfQ = Institute for Quality Assurance; Ig = immunoglobulin; MOG = myelin oligodendrocyte glycoprotein.

The clinical laboratories (Innsbruck, Mayo Clinic, Oxford, and Sydney; centers 1–4) sent the following groups of coded serum samples and clinical information to the Institute for Quality Assurance (IfQ; Lübeck, Germany):

1. Phase I: 89 coded samples sent to centers 1–4 and center 5 (Euroimmun) for testing (figure 1)
  1. MOG-IgG clearly positive: 39 blinded samples from all laboratories with a previously determined clearly positive MOG-Ab serostatus (high titers or fluorescence-activated cell sorting [FACS] binding ratios, supplementary methods, table e-2, links.lww.com/NXI/A189), all of them diagnosed with inflammatory demyelinating diseases known to be associated with MOG-IgG (such as ADEM, aquaporin-4 [AQP4] antibody–negative neuromyelitis optica spectrum disorder (NMOSD), optic neuritis, myelitis, and other demyelinating diseases).
  2. MOG-IgG clearly negative (negative or very low titers or FACS binding ratios, supplementary methods, table e-2, links.lww.com/NXI/A189): 40 blinded samples from all laboratories with a previously determined clearly negative MOG-Ab serostatus. Eighteen of the 40 samples were from people who also presented with clinically overlapping features such as optic neuritis, myelitis, ADEM, or encephalitis. The other samples were from controls (7 from people with MS, 5 from people with other neurologic diseases, and 10 from healthy controls).
  3. Ten technical controls (humanized monoclonal MOG-Ab 8-18-C5,^30^ 5 samples IgG1, and 5 samples IgM (kappa) in different dilutions, but of unknown IgG or IgM concentration, contributed by center 5.
2. Phase II: 100 coded samples sent to 5 centers for testing (18 repeat and 82 new, figure 1)
  1. Nine positive and 9 negative samples from phase I were sent out a second time to assess interassay variations.
  2. Thirty healthy blood donors were contributed by the IfQ. No clinical information was available, and samples were not pretested for antibodies against MOG or other autoantigens.
  3. MOG-IgG low/borderline positive: 39 blinded samples from all laboratories with a previously determined low positive MOG-IgG serostatus (just above the individual cutoff values, supplementary methods, table e-2, links.lww.com/NXI/A189). Thirty-six of these samples were from people with inflammatory demyelinating diseases associated with MOG-IgG and 3 were from patients with MS.
  4. MOG-IgG borderline negative: 13 blinded samples from all laboratories with a previously determined borderline negative MOG-IgG serostatus (just below the individual cutoff values, supplementary methods, table e-2, links.lww.com/NXI/A189). Five of these samples were from patients with inflammatory demyelinating diseases associated with MOG-IgG and 8 were from controls (3 from people with MS and 5 from people with other neurologic diseases).

### Standard protocol approvals, registrations, and patient consents

The present study was approved by the ethical committees of Medical University of Innsbruck (AM3041A and AM4059), Oxford (REC 16/SC/0224), Mayo Clinic (institutional review board 08-007810), and Sydney (NEAF 12/SCHN/395). All samples were anonymized before sending to center IfQ for blinding.

### Laboratory methods and analysis

All samples and controls were recoded, aliquoted, and distributed by an investigator not involved in antibody testing from the IfQ, Lübeck, Germany, to the 5 testing centers, which performed the 7 live CBAs (4 CBA-IF and 3 CBA-FACS), 1 fixed CBA-IF for MOG-IgG, 1 live CBA-IF for MOG-IgM, and 2 ELISAs for MOG-IgG in the 2 study phases (figure 1, table 1 and supplementary methods, links.lww.com/NXI/A189).

**Table 1:**
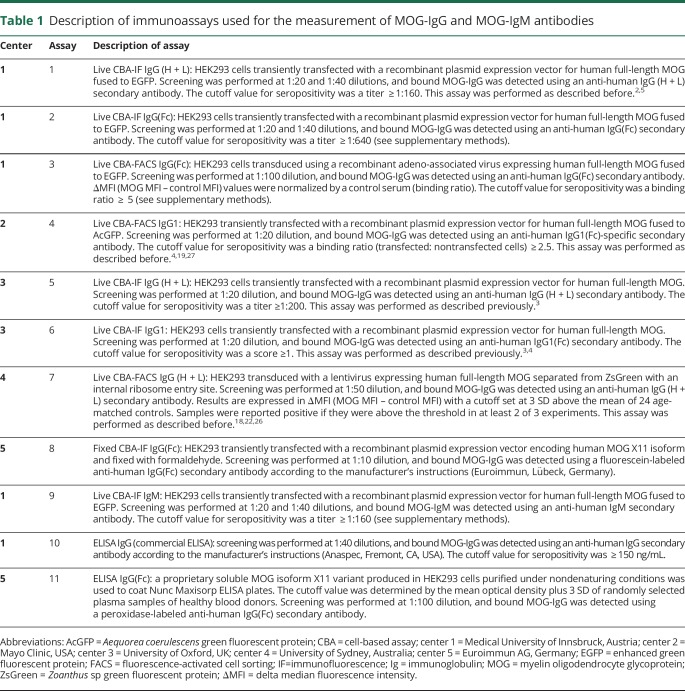
Description of immunoassays used for the measurement of MOG-IgG and MOG-IgM antibodies

### Statistical analysis

Upon completion of the testing, the assay results from each center were entered onto a web-based database. The data were then unblinded and analyzed. Statistical analyses were performed using IBM SPSS software (release 24.0; IBM, Armonk, NY) or GraphPad Prism 8 (GraphPad, San Diego, CA). Correlation of parameters was analyzed with Spearman nonparametric correlation. Kappa statistics were used to assess the concordance between assays. All graphs were created using GraphPad Prism.

### Data availability

The data set used and analyzed during the current study is included in the main text and the supplementary files.

## Results

### CBAs for MOG-IgG show a very good agreement on clear positive and negative samples

**Figure 2:**
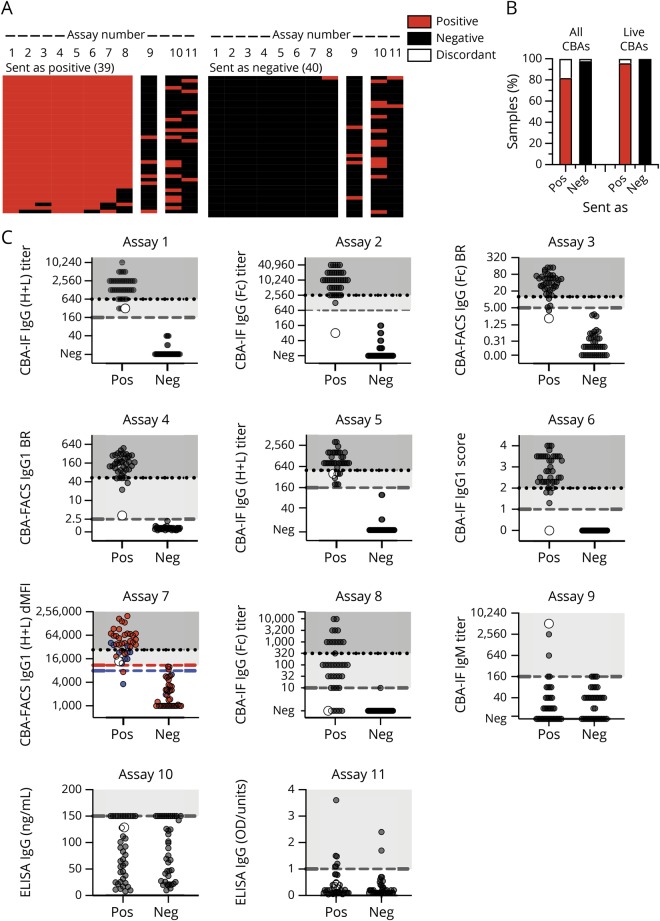
Agreement of MOG-Ab assays on clear positive and negative samples (A) Heatmap of the qualitative results for samples sent as clearly positive (n = 39) or negative (n = 40). Each column is an individual assay (1–8 MOG-IgG CBAs, 9 MOG-IgM CBAs, and 10–11 MOG-IgG ELISAs), and each row is an individual serum sample. Results are based on qualitative results; negative samples are black, and positive samples are red. The samples are shown according to their serostatus sent by the individual centers. (B) Agreement of MOG-IgG CBAs according to the samples sent (pos = positive, neg = negative). Results (in % of all samples) are grouped according to their agreement in all 8 CBAs or in the 7 live CBAs (red: positive in all CBAs, black: negative in all CBAs, white: discordant). (C) Quantitative results for all assays. The cutoff values for all assays except assay Nr. 7 are indicated by the dashed gray lines. For assay Nr. 7, cutoff levels for pediatric samples (blue dots) are indicated by the blue dashed line, and cutoff levels for adult samples (red dots) are indicated by the red dashed line. The quantitative range of each assay result for its probability to be seropositive in all live CBAs is indicated by the dotted line and shaded in darker gray (100% probability), whereas the range of discordant samples is shaded in light gray. A single sample with high IgM titer 1:5120 and low positive in the IgG (H + L) and on IgG1 but not in another IgG1 and the IgG(Fc) assays is indicated by the larger white dot. BR = binding ratio; CBA = cell-based assay; dMFI = delta mean fluorescence intensity; Ig = immunoglobulin; MOG = myelin oligodendrocyte glycoprotein.

In the first phase of this study (figure 1, phase 1), all centers analyzed samples sent as clearly positive (n = 39) or negative (n = 40) by centers 1–4 (figure 2A, table e-3, links.lww.com/NXI/A189). In general, there was a very good agreement for the 8 MOG-IgG CBAs (figure 2B): 39/40 (97.5%) samples sent as negative were negative in all 8 CBAs. This agreement was 100% if the fixed commercial CBA was excluded. For the samples submitted as positive, 32/39 (82%) samples were concordant across all 8 CBAs; again, this improved to 92% (36/39) if the fixed CBA was excluded. Overall, there was 96% concordance across all samples when tested on live platforms in 4 international testing centers. The concordance dropped to 90% if the results of the fixed CBA tested in-house at center 5 were included.

MOG-IgM antibodies at a titer 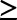 1:160 were a rare finding in samples sent as clear positive (5/39, 13%). One of these 5 samples had a high MOG-IgM titer (1:5,120, figure 2C, assay Nr. 9, large gray dot) and was low positive for MOG-IgG in 4 assays (using IgG (H + L) and IgG1 secondary antibodies), but negative in 4 assays (using IgG(Fc) and IgG1 secondary antibodies). The other 4 samples were positive for MOG-IgG in all CBAs. MOG-IgMs at a titer 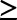
1:160 were absent in all 40 samples sent as negative.

Overall, there was excellent agreement between the 7 live MOG-IgG CBAs (median kappa value 0.975, range 0.924–1.000). The agreement of the fixed MOG-IgG CBA with the live MOG-IgG CBAs was very good (median kappa value 0.822, range 0.821–0.847). There was no agreement between the MOG-IgG and MOG-IgM CBAs (median kappa value −0.003, range −0.180 to 0.103) or between the MOG-IgG CBAs and the ELISA (median kappa value 0.112, range 0.105–0.203).

The quantitative values for all assays are provided in figure 2C. Most of the assays had a very clear separation of positive and negative samples. The quantitative range in which an individual sample is positive in all live CBAs is indicated by the dotted line and shaded in darker gray (100% probability). For example, if a result of assay Nr. 1 is positive with an MOG-IgG titer of 1:1,280, there is 100% probability that this result is also positive in all other live CBAs. However, on the same assay, an MOG-IgG titer of 1:320 (light gray area) is more likely to have discrepant results between centers.

**Figure 3:**
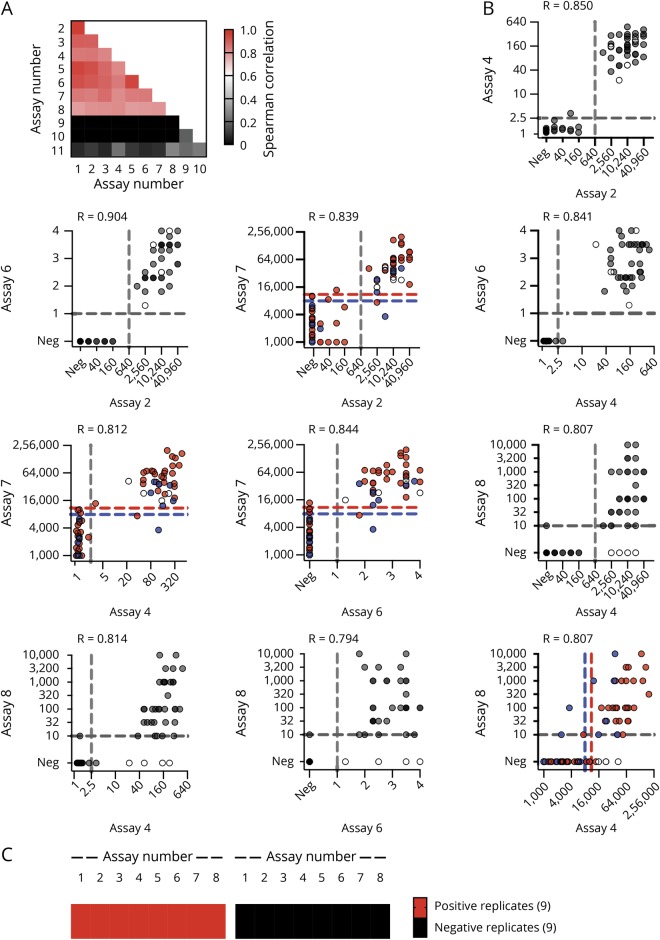
Correlation of MOG-IgG assays and reproducibility of assay results (A) Heatmap of Spearman correlation coefficients for all correlations. (B) Correlation of illustrative live (Nr. 2, 4, 6, and 7) and fixed CBAs (Nr. 8): Nr. 2 (center 1): CBA-IF IgG(Fc) titer (1), Nr. 4 (center 2): CBA-FACS IgG1 binding ratio, Nr. 6 (center 3): CBA-IF IgG1 binding score, Nr. 7 (center 4): CBA-FACS IgG (H + L) delta mean fluorescence intensity, Nr, 8 (center 5): CBA-IF IgG(Fc) titer (1). The cutoff values for all assays except assay Nr. 7 are indicated by the dashed gray lines. For assay Nr. 7, cutoff levels for pediatric samples (blue dots) are indicated by the blue dashed line, and cutoff levels for adult samples (red dots) are indicated by the red dashed line. Samples that are positive in live CBAs but not the fixed CBA (assay Nr. 8) are indicated by the white dots. (C) Qualitative results for 9 positive and 9 negative samples from phase I retested in a blinded way by all assays in phase II. CBA = cell-based assay; IF = immunofluorescence; Ig = immunoglobulin; MOG = myelin oligodendrocyte glycoprotein.

It is evident that there is very good correlation of quantitative results for the 7 live MOG-IgG CBAs (median Spearman correlation coefficient R = 0.866, range 0.806–0.961; figure 3A) and a lower correlation between the fixed CBA and the live MOG-IgG CBAs (median R = 0.800, range 0.778–0.809). There was no correlation between the MOG-IgG and MOG-IgM CBAs (median kappa value −0.071, range −0.134 to 0.179) or the MOG-IgG CBAs with ELISA (median R = 0.094, range 0.060–0.273). These correlations are shown in more details in 1 illustrative assay per center (figure 3B, assays Nr. 2, 4, 6, 7, and 8). Although the separation of negative and positive samples was very good for the live CBAs (assays Nr. 2, 4, 6, and 7), the fixed assay (Nr. 8) was negative for 5 samples clearly positive in the live CBAs.

As a technical control, we included 10 samples containing humanized monoclonal MOG-IgG (5) or MOG-IgM (5) antibodies provided by center 5. Results are shown in supplementary results (figure e-2, links.lww.com/NXI/A189). Importantly, these humanized monoclonal antibodies were not recognized by some of secondary antibodies, particularly the anti-human IgG1 antibody. Moreover, the anti-human IgG (H + L), but not the IgG(Fc) secondary antibodies, also recognized the humanized monoclonal MOG-IgM at the lowest dilution as borderline negative for MOG-IgG.

### MOG-IgG results are reproducible within centers

All centers reproduced the MOG-IgG results from their samples submitted for phase I and the 9 positive and 9 negative replicates that were resent blinded and integrated into the cohort with the borderline samples in phase II (figure 3C and supplementary results, figure e-3 and table e-3, links.lww.com/NXI/A189).

### CBAs for MOG-IgG show less agreement on low positive and borderline negative samples

**Figure 4:**
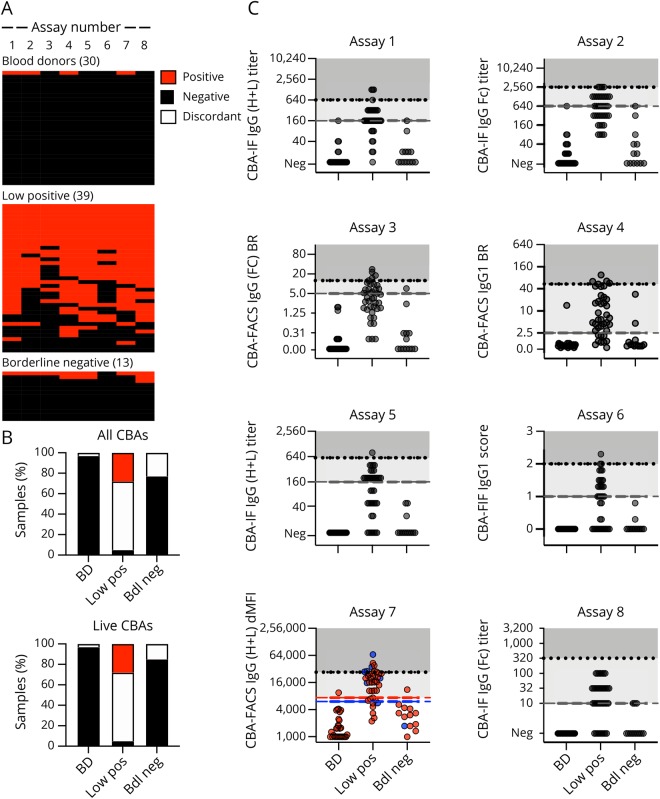
Agreement of MOG-Ab assays on low positive and borderline negative samples and blood donors Qualitative and quantitative results of all MOG-IgG CBAs for blood donors (BD, n = 30), low positive (n = 39), and borderline negative (n = 40) samples. (A) Qualitative results according to the serostatus sent. Each column is an individual MOG-IgG CBA, and each row is an individual serum sample. Negative samples are black, and positive samples are red. The samples are shown according to their serostatus sent by the individual centers. (B) Agreement of assay results for IgG CBAs (assays 1–8). Sample are grouped according to their agreement in all 8 CBAs or in the 7 live CBAs (red: positive in all CBAs, black: negative in all CBAs, white: discordant). (C) Quantitative results for the 8 MOG-IgG CBA-IF and CBA-FACS assays. The cutoff values for all assays except assay Nr. 7 are indicated by the dashed gray lines. For assay Nr. 7, cutoff levels for pediatric samples (blue dots) are indicated by the blue dashed line, and cutoff levels for adult samples (red dots) are indicated by the red dashed line. The quantitative range of each assay result for its probability to be seropositive in all live CBAs is indicated by the dotted line and shaded in darker gray (100% probability), whereas the range of discordant samples is shaded in light gray. BD = blood donor; bdl neg = borderline negative values just below the individual cutoff levels; CBA = cell-based assay; FACS = fluorescence-activated cell sorting; IF = immunofluorescence; Ig = immunoglobulin; low pos = positive values just above the individual cutoff levels; MOG = myelin oligodendrocyte glycoprotein.

In the second phase of this study (figure 1, phase II), we analyzed samples sent as low positive (n = 39) or borderline negative (n = 13) by the participating centers and 30 samples from healthy blood donors. Qualitative results obtained by the different CBAs for MOG-IgG are shown in figure 4A and supplementary results, table e-3, links.lww.com/NXI/A189. In general, there was a good agreement for the 8 MOG-IgG CBAs for the samples from blood donors: 29 of the 30 samples (97%) were negative in all 8 CBAs, and 1 sample was positive in 4 CBAs (figure 4B).

The agreement for low positive samples was less good: 2 of the 39 samples (5%) sent as borderline positive were negative in all 8 CBAs, and only 11 samples (28%) were positive in all 8 CBAs. Therefore, the CBAs had a complete agreement of 33%. The remaining 26 samples (67%) were positive in 7 (n = 8), 6 (n = 2), 5 (n = 6), 4 (n = 2), 3 (n = 3), 2 (n = 2), and 1 (n = 3) assays. The agreement for borderline negative samples was better: 10 of the 13 samples (77%) were negative in all 8 CBAs, and no sample (0%) was positive in all 8 CBAs. Therefore, the CBAs had a complete agreement of 77%. The remaining 3 samples (23%) were positive in 7 (n = 1), 3 (n = 1), and 1 (n = 1) assays.

The quantitative values for all MOG-IgG CBAs are provided in figure 4C. From this figure, it is evident that many of the positive signals are around the cutoff and under the quantitative range described above for samples positive in all live CBAs.

Furthermore, MOG-IgMs at a titer 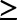
1:160 were a rare finding in samples sent as low positive (n = 5, all 1:160) and absent in samples sent as borderline negative or blood donors. These 5 samples were positive for MOG-IgG in 1 (n = 1), 4 (n = 1), 5 (=2), and 6 (n = 1) CBAs.

### Samples identified as MOG-IgG positive in all CBAs are associated with a non-MS demyelinating disease course

None of the 13 patients with clinically definite MS in the study or the 5 patients with other neurologic diseases were within the 47 patients who tested positive on all live CBAs (supplementary results, figure e-4, links.lww.com/NXI/A189). These 47 patients had typical MOG-IgG-associated clinical phenotypes such as optic neuritis, ADEM, myelitis, AQP4-seronegative NMOSD, or other demyelinating phenotypes reported to be associated with MOG-IgG. The 32 discordant samples were from 27 patients with typical MOG-associated clinical phenotypes mentioned above, but also from a healthy blood donor and 4 patients with MS. Finally, the 82 samples negative in all live CBAs were from 24 patients with typical MOG-associated clinical phenotypes, MS (9), other neurologic diseases (10), and healthy controls (39).

## Discussion

In this study, we compared the reproducibility among the most frequently used assays for serum MOG-IgG detection, such as live and fixed CBA-IF, live CBA-FACS, and ELISA. Our data clearly indicate that strong positive and clearly negative samples are reproducible between centers where live cells expressing native full-length human MOG are used as the assay substrate. In the 4 different national testing centers using different live CBAs, there was 96% concordance for all samples tested. The agreement was less good when a fixed CBA-IF (90%) tested in-house by the company (center 5) was included, which is consistent with recent studies demonstrating that some conformational epitopes of MOG are lost upon fixation of MOG-expressing cells.^4,16,26^ Importantly, most of these discordant negative results on the fixed MOG-IgG assay had high MOG-IgG titers in live CBAs and were from typical MOG-IgG-associated demyelinating syndromes. There is utility in the commercial fixed MOG-IgG testing in places where live MOG-IgG CBAs are unavailable, but this assay will miss 10–15% of positive cases. A recent study highlighted an issue with specificity in commercial MOG-IgG testing, particularly in samples that were only positive at low dilutions.^4^ Therefore, clinicians should consider retesting unexpected MOG-IgG results at centers offering live CBAs.

Finally, ELISAs did not distinguish between the positive and negative patient samples and showed no concordance with CBAs for detection of human MOG-IgG conclusively demonstrating that ELISAs are not suitable for the detection of human MOG-IgG. Although this has been shown in several studies (summarized in ref 28), some laboratories still use this method for MOG-IgG detection. We hope that our findings inform neurologists that only CBAs should be used for the measurement of human MOG-IgG. Moreover, and in agreement with previous studies,^4,16,26^ live CBAs remain the gold standard for the detection of human MOG-IgG.

The agreement of MOG-IgG CBAs for low positive sample was less good (33% concordance), and MOG-IgG assays were particularly discordant at the borderline of positivity. This raises the pertinent question where to draw cutoff values and how they influence the clinical interpretation of diagnostic results. If we examine the clinical phenotype of people with high MOG-IgG levels, which are consistently detected by all CBAs, we identify patients with non-MS demyelinating phenotypes (such as ADEM, NMOSD, optic neuritis, myelitis, and other demyelinating diseases).^1^ In contrast, the low positive samples ,which showed a lack of reproducibility between centers, had a wider range of clinical phenotypes that mostly include the same phenotypes (ADEM, NMOSD, optic neuritis, myelitis, and other demyelinating diseases), but also a proportion of every control group (clinically definite MS, other neurologic diseases, and healthy individuals), making their interpretation difficult. It is unlikely that lower levels of pathogenic antibody cause a wider disease presentation, suggesting that some of these phenotypes are not driven by MOG-IgG. Hence, an argument can be made that the presence of low positive MOG-IgG is only meaningful in the correct clinical context such as in patients with ON, myelitis, ADEM, or encephalitis but not in the context of other diseases, particularly MS.^1,19,31^ This is a circular but reasonable interpretation of low positive results, but with caveats. There will be a false-positive rate even within the correct clinical context that should be considered, and an estimate of this would be useful for any test. Second, clinical criteria are not perfect. There are individuals who fulfill criteria for MS, but are often atypical; perhaps the MOG-IgG result has utility in this context in ruling out MS and should not be ignored out of hand. Importantly, when extrapolating from experiences on the treatment of NMOSD and a recent larger study on treatment of patients with MOG-IgG from France, disease-modifying treatments for MS may not work in MOG-IgG-positive patients and may even exacerbate disease.^1,31–33^ The third interpretation is that these low positive results that are not reproducible between centers are not useful clinically and in fact dilute the utility of a more specific test. Finally, as a general consideration in samples not taken at disease onset, other confounding factors such as preceding steroid use or other immunosuppressive treatments and remission could lower a positive MOG-IgG result. It is important to note that MOG-IgG levels are often non-normally distributed in large patient cohorts, and a skewing toward these lower MOG-IgG titers has been observed in many studies.^1^

Samples scored as low positive for MOG antibodies are much less concordant than the clearly positive samples across the 7 live MOG assays (28% vs 92%). Importantly, the MOG-IgG low positives are also less concordant than low positive samples in similar assays for other autoantigens such as AQP4. In 2016, we published a European multicenter validation experiment comparing 21 AQP4-IgG assays.^34^ In this study 5 live CBAs (3 CBA-IF and 2 live CBA-FACS) had sensitivities, specificities, and accuracies greater than 90% similar to the 7 live MOG-IgG CBAs in our current study. These 5 AQP4-IgG assays were compared on 66 AQP4-Ab-positive samples, thereof 52 were high positive (median semiquantitative score in the live CBAs 2.5–4) and 14 low positive (median semiquantitative score in the live CBAs 1–2).^34^ The agreement for high positive AQP4-IgG samples across all 5 live CBAs was 52/52 (100%), similar to the strong positives for the MOG-IgG tests that were 92% concordant (36/39). Only 11/39 (28%) of the low positive MOG-IgG samples were concordant on all 7 MOG CBAs in sharp contrast to the low positive AQP4-IgG samples that remained strongly concordant across tests: 11/14 (79%) were positive on all 5 tests, and the 3 discordant samples were positive on 4/5 assays. Thus, the confidence in the reproducibility of a low positive result between centers by live CBA for MOG-IgG is much lower than that of a low positive AQP4-Ab result.

Overall, only 10 samples were MOG-IgG and MOG-IgM double positive: 4/47 (9%) were positive by all 7 live CBAs, all of them with non-MS demyelinating syndromes, and 6/32 (19%) had discordant results, 5 of them with non-MS demyelinating syndromes and 1 with MS. This frequency of double-positive patients is comparable to findings recently reported by Pedreno et al.^35^ (11/97, 11%) and Tea et al.^26^ (13/281, 5%). In addition, MOG-IgM antibodies were found in 4/82 (5%) samples identified as seronegative by all 7 live CBAs. These included 1 patient with non-MS demyelinating syndrome, 1 with MS, and 2 healthy controls. These data do not demonstrate a clinical utility for the detection of MOG-IgM antibodies.

Further work is now needed to better define the most useful clinical cutoff and to establish whether there is any added benefit in identifying patients with low positive MOG-IgG. We propose that this should be done in a collaborative effort. We need to better characterize false-positive cases, such as classical MS cases, other neurologic diseases, and healthy individuals, and get more information on the clinical sensitivity and specificity of all assays by using appropriate controls, such as systemic autoimmune diseases, noninflammatory neurologic controls, and healthy controls. It is of great interest to establish how these antibodies relate to different clinical phenotypes and whether they are a mixture of pathogenic and bystander antibodies that all bind MOG in vitro.

To conclude, we have shown that currently used live CBAs to measure MOG-Abs showed excellent agreement for clearly positive and negative samples, but low positive samples were more discordant. Further work is now required to standardize the clinically most useful assay.

## Glossary

ADEM: acute disseminated encephalomyelitis
AQP4: aquaporin-4
CBA: cell-based assay
FACS: fluorescence-activated cell sorting
IF: immunofluorescence
IfQ: Institute for Quality Assurance
Ig: immunoglobulin
MOG: myelin oligodendrocyte glycoprotein
NMOSD: neuromyelitis optica spectrum disorder
